# Инфицированность SARS-CoV-2 в зависимости от уровня обеспеченности витамином D

**DOI:** 10.14341/probl12820

**Published:** 2021-10-06

**Authors:** Т. Л. Каронова, А. Т. Андреева, К. А. Головатюк, Е. С. Быкова, И. И. Скибо, Е. Н. Гринева, Е. В. Шляхто

**Affiliations:** Национальный медицинский исследовательский центр им. В.А. Алмазова; Национальный медицинский исследовательский центр им. В.А. Алмазова; Национальный медицинский исследовательский центр им. В.А. Алмазова; Национальный медицинский исследовательский центр им. В.А. Алмазова; ООО «НПФ «ХЕЛИКС»; Национальный медицинский исследовательский центр им. В.А. Алмазова; Национальный медицинский исследовательский центр им. В.А. Алмазова

**Keywords:** дефицит витамина D, 25(OH)D, SARS-CoV-2, COVID-19, ПЦР-тест

## Abstract

ОБОСНОВАНИЕ. В настоящее время во всем мире активно обсуждается ассоциация между дефицитом витамина D и степенью тяжести течения COVID-19.ЦЕЛЬ. Целью настоящей работы было оценить распространенность недостатка и дефицита витамина D и сопоставить с показателями инфицированности SARS-CoV-2 в восьми федеральных округах РФ.МАТЕРИАЛЫ И МЕТОДЫ. В анализ включены результаты обследования 304 564 лиц (234 716 женщин; 77,1%), у которых были известны показатели концентрации 25(ОН)D в сыворотке крови в период с сентября 2019 по октябрь 2020 г.РЕЗУЛЬТАТЫ. Лишь 112 877 человек (37,1%) имели нормальный уровень 25(ОН)D в сыворотке крови, остальные находились в недостатке или дефиците. Недостаток и дефицит витамина D были представлены с одинаковой частотой у женщин и мужчин, также не было выявлено различий в зависимости от географического расположения субъектов РФ и возраста у лиц от 18 до 74 лет. Однако лица старше 75 лет чаще имели дефицит витамина D, в то время как лица моложе 18 лет в более 50% случаев имели нормальный его уровень. У 21 506 больных было выполнено исследование на SARS-CoV-2 методом полимеразной цепной реакции (ПЦР), результаты которого сопоставлены с уровнем обеспеченности витамином D. Положительный результат ПЦР был выявлен у 3193 обследованных, отрицательный — у 18 313. Не выявлено различий в инфицированности пациентов в условиях дефицита и нормального уровня обеспеченности витамином D. Так, при уровне 25(ОН)D ниже 20 нг/мл (4978 тестов) количество положительных ПЦР-тестов составило 14,8%, при уровне 20–30 нг/мл (7542 тестов) — 14,9%, 30–50 нг/мл (6622 тестов) — 15,0% и при значении более 50 нг/мл (4612 тестов) — 13,9%.ЗАКЛЮЧЕНИЕ. Таким образом, не выявлено зависимости между уровнем обеспеченности витамином D и числом положительных ПЦР-тестов к SARS-Co-2 ни в одном из регионов проживания, что свидетельствует об отсутствии связи между инфицированностью COVID-19 в РФ и уровнем обеспеченности витамином D, хотя дефицит нутриента сохраняется во всех регионах и наиболее часто диагностируется у лиц старше 75 лет.

## Abstract (en)

BACKGROUND: The association between vitamin D deficiency and the severity of COVID-19 is currently being actively discussed around the world.AIM: The aim of this study was to assess the prevalence of vitamin D insufficiency and deficiency and compare it with the incidence rates of SARS-CoV-2 in eight Federal Districts of the Russian Federation.MATERIALS AND METHODS: We included 304,564 patients (234,716 women; 77,1%) with serum 25(OH)D levels results performed September 2019 through October 2020.RESULTS: Only 112,877 people (37.1%) had a normal serum 25(OH)D level, others had a deficiency. Vitamin D insufficiency and deficiency was presented with the same frequency in women and men, and no differences were found depending on the geographical location and age in subjects from 18 to 74 years old. However, subjects over 75 years more often had vitamin D deficiency, while subjects under 18 years had normal levels in over 50% cases. In addition, 21,506 patients were tested for SARS-CoV-2 by PCR with further comparison of results with serum 25(OH)D level. The SARS-CoV-2 positivity rate was detected in 3,193 subjects, negative in 18,313. There were no differences in the morbidity in a vitamin D deficiency and a normal level. Thus, 14.8% subjects had positive PCR rates among vitamin D deficiency patients (4,978 tests), 14.9% when 25(OD)D level was from 20 to 30 ng/ml (7,542 tests), 15.0% among those who had 25(OH)D 30- 50 ng/ml (6,622 tests), and 13.9% when vitamin D was more than 50 ng/ml (4,612 tests).CONCLUSION: There was no association between the COVID-19 incidence and vitamin D status in different regions of Russia. Although the nutrient deficiency persists in all regions and is most often diagnosed in people over 75 years old.

## ОБОСНОВАНИЕ

Как известно, дефицит витамина D остается значимой медико-социальной проблемой для многих стран вне зависимости от географического расположения [1–3]. Отечественные исследования последних лет подтвердили данные о сохраняющейся высокой распространенности недостатка и дефицита витамина D как в северных, так и в южных регионах РФ [4][5]. Так, анализ результатов первого этапа многоцентрового исследования, проведенного в 10 регионах РФ весной 2020 г., показал, что среди 445 участников исследования в возрасте от 18 до 50 лет, не принимавших ранее препараты витамина D в качестве монотерапии или в комбинации с препаратами кальция, уровень 25(ОН)D в сыворотке крови более 30 нг/мл был диагностирован только у 15,73% обследованных, в то время как доля лиц с недостатком и дефицитом составила 84,27% [5]. Сравнивая полученные авторами данные с ранее опубликованными [4], хочется отметить тот факт, что в РФ сохраняется высокая распространенность недостатка и дефицита витамина D, и доля лиц с нормальным уровнем обеспеченности не превышает 20%. Учитывая известные плейотропные эффекты витамина D, сегодня активно изучается вопрос о возможном вкладе данного нутриента в профилактику и лечение острых респираторных вирусных инфекций, включая новую коронавирусную инфекцию COVID-19 [6–9].

При анализе публикаций на 1 мая 2021 г. было найдено более 90 статей и обзоров, посвященных изучению связи между низким уровнем 25(ОН)D в крови и инфицированностью, степенью тяжести и исходами COVID-19 [10]. Следует отметить тот факт, что большинство публикаций носит описательный характер и представлено в основном обзорами и метаанализами, и лишь небольшая часть работ относится к оригинальным исследованиям с демонстрацией собственных данных.

Данные современной литературы свидетельствуют о наличии так называемых иммуномодулирующих свойств витамина D [11][12], что сегодня подтверждает активное его участие как в клеточном, так и гуморальном иммунном ответе [13–15]. Так, опубликованные результаты анализа продемонстрировали более частое обнаружение положительного результата теста методом полимеразной цепной реакции (ПЦР) к SARS-CoV-2 у лиц с дефицитом витамина D по сравнению с теми, чей уровень 25(ОН)D крови превышал 30 нг/мл и соответствовал норме [16], а также снижение степени тяжести коронавирусной инфекции при более высоком уровне 25(ОН)D и уменьшение длительности госпитализации на фоне приема препаратов витамина D [17][18]. Отечественные исследования в этой области малочисленны, чем и обусловлен интерес исследователей к этой теме.

## ЦЕЛЬ ИССЛЕДОВАНИЯ

Уточнить распространенность недостатка и дефицита витамина D и сопоставить с показателями инфицированности SARS-CoV-2 среди лиц с уровнем обеспеченности витамином D, проживающих в различных федеральных округах РФ.

## МАТЕРИАЛЫ И МЕТОДЫ

Место и время проведения исследования

Место проведения. ФГБУ «Национальный медицинский исследовательский центр им. В.А. Алмазова» МЗ РФ, г. Санкт-Петербург, Россия.

Время исследования. В период с сентября 2019 по октябрь 2020 г. Статистическая обработка данных проведена в период с 01.02.2021 по 31.05.2021 г.

Для оценки численности населения использованы данные Росстата на 01.02.2020 г. [19], для уточнения количества заболевших и умерших от COVID-19 — данные сайта Минздрава [20]. Приведенные в статье данные проанализированы на момент 25.02.2021 г.

Изучаемая популяция

Популяция. Исследовали уровни 25(ОН)D в сыворотке крови и результаты ПЦР-тестов на SARS-CoV-2/SARS-CoV 304 564 пациентов, проживающих в восьми федеральных округах (ФО) РФ (234 716 женщин; 77,1%), обратившихся в ООО «НПФ «ХЕЛИКС».

Критерии включения: мужчины и женщины 18 лет и старше, обратившиеся для исследования уровня 25(ОН)D в ООО «НПФ «ХЕЛИКС» в период с сентября 2019 по октябрь 2020 г.

Критерии исключения: не предусмотрены.

Способ формирования выборки из изучаемой популяции (или нескольких выборок из нескольких изучаемых популяций)

Из базы данных ООО «НПФ «ХЕЛИКС» были отобраны результаты пациентов, имевших хотя бы одно определение 25(ОН)D в сыворотке крови в период с сентября 2019 по октябрь 2020 г. Дополнительно из 304 564 пациентов были отобраны данные 32 197 пациентов, имевших по крайней мере один результат ПЦР-теста на SARS-CoV-2 за указанный период. При наличии хотя бы одного положительного (+) результата ПЦР-теста пациент считался инфицированным SARS-CoV-2. При наличии нескольких результатов исследования уровня 25(ОН)D у одного и того же пациента для анализа использовался результат, наиболее близкий к дате проведения исследования ПЦР.

Дизайн исследования

Исследование выполнено в дизайне случай-контроль. Данные пациентов предоставлены ООО «НПФ «ХЕЛИКС» в заслепленном виде.

Методы

Уровень 25(ОН)D был определен методом хемилюминесцентного иммуноанализа на анализаторе Unicel DxI800 (Beckman Coulter, США) с использованием систем UniCel DxI. Согласно рекомендациям Российской ассоциации эндокринологов 2015 г., за нормальный уровень обеспеченности витамином D принималось значение 25(ОН)D в сыворотке крови ≥30 нг/мл (≥75 нмоль/л), за недостаточность — ≥20 и <30 нг/мл (≥50 и <75 нмоль/л), за дефицит — <20 нг/мл (<50 нмоль/л) и за тяжелый дефицит витамина D — менее 10 нг/мл (<25 нмоль/л). Диапазон определения 25(ОН)D составил 4,4–210,0 нг/мл.

Выявление РНК коронавируса SARS-CoV-2 проводили методом обратной транскрипции и ПЦР в режиме реального времени (SARS-CoV-2/SARS-CoV) по ТУ 21.20.23-116-46482062-2020 с использованием амплификатора детектирующего «ДТ Прайм» (ООО «НПО ДНК-Технология») и наборов для выявления РНК коронавирусов (производитель ООО «ДНК-Технология ТС»).

Статистический анализ

Статистический анализ данных выполнен с помощью программного комплекта IBM SPSS Statistics for Windows ver. 26 (IBM Corp., Armonk, N.Y., USA).

Этическая экспертиза

Протокол исследования Вер. 1.1 от 23.10.2020 г. был одобрен этическим комитетом ФГБУ «Национальный медицинский исследовательский центр им. В.А. Алмазова» МЗ РФ 30 ноября 2020 г. (выписка № 1011-20-02С).

## РЕЗУЛЬТАТЫ

Среди всех обследованных (304 564 человек) в ООО «НПФ ХЕЛИКС» в период с сентября 2019 по октябрь 2020 г. и имеющих результаты исследования уровня 25(ОН)D крови лица в возрасте до 18 лет составили 45 811 (15,0%) (23 617 женщин; 51,6%). Доля лиц в возрасте от 18 до 44 лет была значительно выше — 154 310 (50,7%) (126 076 женщин; 81,7%). В возрасте 45–60 лет обследованы 62 853 (20,6%) (50 635 женщин; 80,6%), в возрасте 61–74 года — 34 331 (11,3%) (28 576 женщин; 83,2%) и старше 75 лет — 7259 человек (2,4%) (5812 женщин; 80,1%). Результаты обследования показали, что большинство обратившихся для определения уровня 25(ОН)D в крови составили женщины независимо от возраста. Дефицит витамина D был обнаружен у 88 427 из 304 564 человек (29,0%), недостаток — 103 260 человек (33,9%). Таким образом, несмотря на активную профилактику недостатка и дефицита витамина D, проводимую в РФ на протяжении последних пяти лет, лишь 112 877 человек (37,1%) имели нормальный уровень 25(ОН)D в сыворотке крови, а остальные находились в условиях недостатка или дефицита витамина D.

Результаты статистического анализа показали, что дефицит витамина D был представлен с одинаковой частотой как у женщин, так и у мужчин и составил 29,5 и 27,5% соответственно (p>0,05). Аналогичные результаты были получены и по встречаемости недостатка витамина D, который был обнаружен у 34,0% женщин и 33,6% мужчин (p>0,05). Таким образом, мы не выявили гендерных различий во встречаемости недостатка и дефицита витамина D в период с сентября 2019 по октябрь 2020 г.

В зависимости от возраста нами установлено, что среди лиц моложе 18 лет дефицит витамина D был диагностирован у 22,3% человек, недостаток — у 31,9%, среди лиц в возрасте 18–44 лет эти показатели соответственно составили 29,2 и 34,1%, среди лиц 45–60 лет — 30,0 и 35,2%, среди лиц в возрасте 61–74 лет — 32,6 и 34,8%. В группе старше 75 лет дефицит и недостаток были обнаружены в 42,2 и 27,2% случаев. Таким образом, различий по встречаемости недостатка и дефицита витамина D у лиц в возрасте от 18 до 74 лет получено не было, в то время как у обследованных в возрасте до 18 лет почти половина имели нормальный уровень 25(ОН)D, а среди лиц старше 75 лет большее количество обследованных имели дефицит витамина D (рис. 1).

Необходимо отметить тот факт, что среди лиц как с нормальным уровнем 25(ОН)D крови, так и с низкой концентрацией, вероятно, были пациенты, принимающие препараты витамина D, однако их долю среди общей обследованной популяции установить в рамках проведенного исследования не представлялось возможным.

При анализе показателей 25(ОН)D в зависимости от региона проживания было установлено, что дефицит и недостаток витамина D среди 121 жителя Дальневосточного ФО имели 62 человека (51,2%), среди 23 894 человек Приволжского ФО — 14 378 (60,2%), среди 95 147 жителей Северо-Западного ФО — 58 566 (61,6%), среди 10 948 жителей Сибирского ФО — 6466 (59,1%), среди 43 482 жителя Уральского ФО — 28 246 (65,0%), среди 60 518 жителей Центрального ФО — 37 822 (62,5%), среди 70 454 человек-жителей Южного ФО — 46 147 (66,5%) и среди 1394 жителей Северо-Кавказского ФО — 921 (66,1%). Таким образом, практически во всех регионах более 60% обследованных имели недостаток или дефицит витамина D, что несколько ниже ранее полученных данных. Однако, учитывая тот факт, что недостаток или дефицит мог быть диагностирован и у лиц, получавших препараты витамины D, полученные данные свидетельствуют об отсутствии достижения целевого уровня 25(ОН)D в сыворотке крови у большинства обследованных независимо от региона проживания (рис. 2).

Среди 304 564 пациентов, имевших данные о концентрации 25(ОН)D в сыворотке крови, 32 197 с целью диагностики SARS-CoV-2 выполнили обследование в период с 26.03.2020 по 31.10.2020 г. и имели данные ПЦР-тестов. После исключения повторных тестов у одного и того же пациента статистическому анализу стали доступны данные 21 506 тестов, из которых хотя бы один положительный результат был выявлен у 3193 обследованных, а в 18 313 случаях результаты теста были отрицательными. Результаты ПЦР-теста были сопоставлены с уровнем обеспеченности витамином D у жителей различных регионов. В том случае, если пациент имел более одного результата 25(ОН)D в сыворотке крови, для статистического анализа использовали данные, максимально приближенные к дате выполнения ПЦР-теста.

Среди обследованных, имевших положительный результат ПЦР-теста, уровень 25(ОН)D в сыворотке крови, а также встречаемость недостатка и дефицита витамина D были аналогичными по сравнению с данными показателями у лиц с отрицательным результатом ПЦР-теста (табл. 1).

Дополнительно в рамках исследования были проанализированы данные Росстата о численности населения, а также Минздрава РФ о количестве случаев заболевших COVID-19 и умерших в результате коронавирусной инфекции. Выявлено, что на 25.02.2021 г. количество случаев COVID-19 было несколько выше в Северо-Западном и Центральном ФО, а число умерших по причине COVID-19 находилось в диапазоне от 1,3% (Дальневосточный ФО) до 3,2% (Южный ФО) (табл. 2).

На основании известных данных о численности населения, числе заболевших СOVID-19 и умерших нами были рассчитаны показатели инфицированности и летальности в различных ФО на 25.02.2021 г. (табл. 2, рис. 3).

Как видно из данных, представленных выше, наибольшее количество выявленных случаев COVID-19 было зарегистрировано в Северо-Западном и Центральном ФО, а показатель летальности от COVID-19 был несколько выше в Сибирском и Южном ФО.

При анализе количества положительных ПЦР-тестов в зависимости от уровня 25(ОН)D в сыворотке крови нами не выявлено различий инфицирования в условиях дефицита и нормального уровня обеспеченности витамином D. Так, при уровне 25(ОН)D в сыворотке крови ниже 20 нг/мл количество положительных ПЦР-тестов составило 14,8% из 4978 тестов, выполненных при дефиците витамина D; при значениях от 20 до 30 нг/мл — 14,9% из 7542 тестов; в диапазоне 30–50 нг/мл — 15,0% из 6622 тестов, а при концентрации более 50 нг/мл — 13,9% из 4612 тестов.

Дополнительно нами было проанализировано количество положительных результатов ПЦР-теста (%) в зависимости от региона проживания и уровня обеспеченности витамином D этих лиц. Из всех регионов только данные из Дальневосточного ФО не подлежали анализу из-за малой выборки, а в остальных регионах достоверных различий получено не было (табл. 3).

Как видно из представленных данных, от 33,6 до 51,4% больных с положительным результатом ПЦР к SARS-CoV-2 имели нормальный уровень обеспеченности витамином D, в то время как у 36,4–48,6% при показателе 25(ОН)D более 30 нг/мл ПЦР тест был отрицательным. Обращает на себя внимание лишь Северо-Кавказский ФО, где отмечалось наибольшее количество лиц с положительным ПЦР-тестом, имеющих дефицит витамина D (37,6%). Однако такая пропорция с преобладанием дефицитных пациентов наблюдалась и среди лиц с отрицательным результатом. Более детальный анализ также не выявил зависимости между суммарным количеством инфицированных и уровнем 25(ОН)D в сыворотке крови (рис. 4).

Таким образом, результаты проведенного исследования еще раз подтвердили высокую распространенность недостатка и дефицита витамина D у жителей различных регионов и не выявили зависимости между инфицированностью COVID-19 и уровнем обеспеченности витамином D в южных, центральных и северных регионах РФ.

**Figure:**
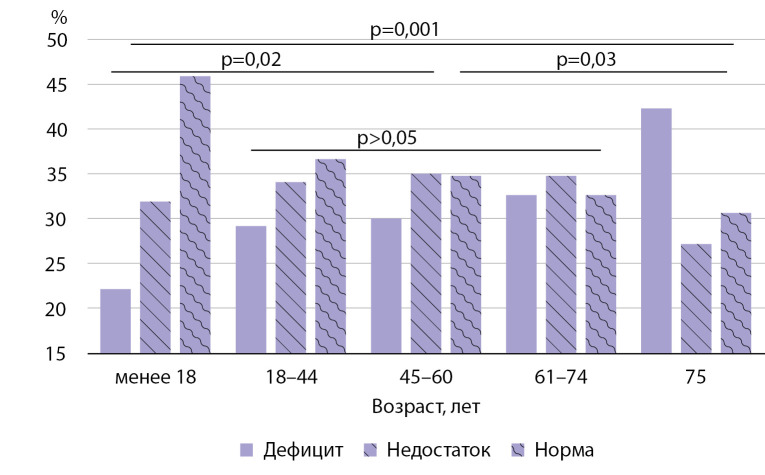
Рисунок 1. Распространенность дефицита витамина D в зависимости от возраста.

**Table:** Таблица 1. Уровень 25(ОН)D и встречаемость дефицита витамина D у лиц с положительным тестом полимеразной цепной реакции к SARS-CoV-2

Параметр	Всеn=21 506	ПЦР (+)n=3193	ПЦР (-)n=18 313	p
25(OH)D, нг/млMinMaxCреднее±SDМедиана, Me[Q25; Q75]	3,22210,531,24±16,6927,41[ 20,54; 37,3]	4,24159,5230,96±16,3527,29[ 20,49; 36,68]	3,22210,531,29±16,7527,43[ 20,55; 37,41]	>0,05
Статус витамина D:Норма, n (%)Недостаток, n (%)Дефицит, n (%),включая тяжелый дефицит (<10 нг/мл), n(%)	8 988 (41,8)7 542 (35,1)4 976 (23,1)356 (1,7)	1 337 (41,9)1 120 (35,0)736 (23,1)45 (1,4)	7 651 (41,8)6 422 (35,0)4 240 (23,2)311 (1,7)	>0,05

 

**Figure:**
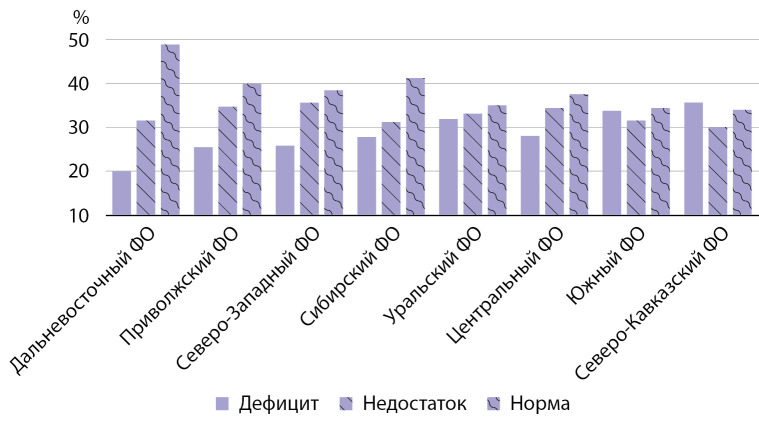
Рисунок 2. Распространенность дефицита витамина D в зависимости от региона проживания.

**Table:** Таблица 2. Количество заболевших и умерших от COVID-19 в зависимости от региона проживания (данные на 25.02.2021 г.)

Федеральный округ	Численность населения (данные Росстата)	Выявлено случаев COVID-19n	Инфицированность (человек на 1000 жителей)	УмерлиотCOVID-19n	Летальность (%)
Дальневосточный	8 131 555	253 091	31,12	3261	1,3
Приволжский	29 087 997	518 761	17,83	10 231	2,0
Северо-Западный	13 952 964	704 649	50,50	15 363	2,2
Сибирский	17 009 249	349 461	20,55	10 180	2,9
Уральский	12 333 234	262 701	21,30	4986	1,9
Центральный	39 251 953	1 661 777	42,34	28 868	1,7
Южный	16 498 642	255 573	15,49	8081	3,2
Северо-Кавказский	9 967 301	163 043	16,36	3435	2,1
ИТОГО	146 232 895	4 169 056	28,51	80 970	1,9

**Figure:**
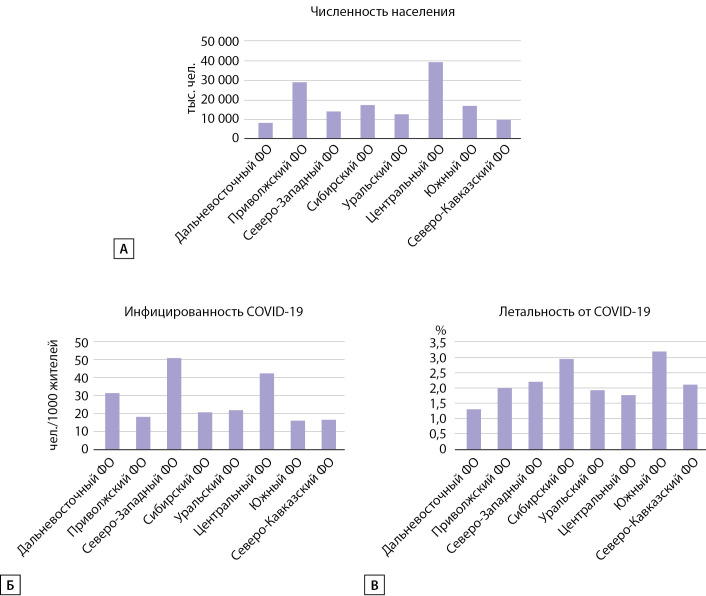
Рисунок 3. Показатели численности населения (А), инфицированности (Б) и летальности (В) от COVID-19 на 25.02.2021 г.,адаптировано с сайта Минздрава РФ (www.стопкоронавирус.рф)

**Table:** Таблица 3. Распределение обследованных в зависимости от уровня обеспеченности витамином D и результатов ПЦР-тестов (результаты представлены по регионам)

Федеральный округ	ПЦР (+)n=3193	Уровень обеспеченности витамином D	ПЦР (-)n=18 313	Уровень обеспеченности витамином D
нормаn=1337	недостатокn=1120	дефицитn=736	нормаn=7651	недостатокn=6422	дефицитn=4240
Дальневосточный, n (%)	2(16,7)	1(50,0)	1(50,0)	0	12(85,7)	4(33,3)	4(33,3)	4(33,3)
Приволжский,n (%)	81(24,1)	39(48,1)	30(37,0)	12(14,8)	336 (80,6)	167(49,7)	98(29,2)	71(21,1)
Северо-Западный, n (%)	1677(15,7)	716(42,7)	607(36,2)	354(21,1)	10 713(86,5)	4411(41,2)	3857(36,0)	2445(22,8)
Сибирский,n (%)	74(30,2)	38(51,4)	23(31,1)	13(17,6)	245(76,8)	119(48,6)	75(30,6)	51(20,8)
Уральский,n (%)	602(20,8)	236(39,2)	222(36,9)	144(23,9)	2897(82,8)	1150(39,7)	1045(36,1)	702(24,2)
Центральный,n (%)	360(13,8)	165(45,8)	113(31,4)	82(22,8)	2610 (87,9)	1231(47,2)	835(32,0)	544(20,8)
Южный,n (%)	102(25,4)	43(42,2)	39(38,2)	20(19,6)	401(79,7)	169(42,1)	171(42,6)	61(15,2)
Северо-Кавказский, n (%)	295(26,8)	99(33,6)	85(28,8)	111(37,6)	1099(78,8)	400(36,4)	337(30,7)	362(32,9)

**Figure:**
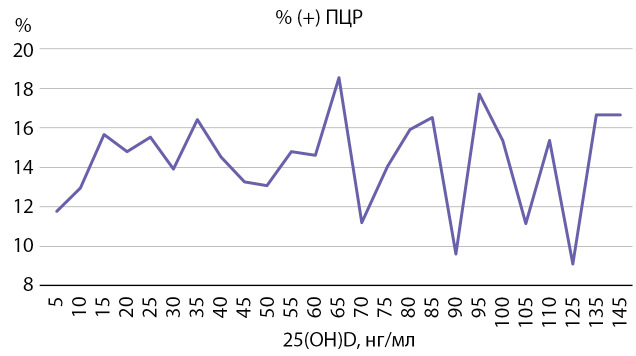
Рисунок 4. Процент положительных ПЦР-тестов при различном уровне 25(OH)D в сыворотке крови.

## ОБСУЖДЕНИЕ

Репрезентативность выборок

Пандемия новой коронавирусной инфекции (COVID-19), вызванная SARS-CoV-2, в настоящее время представляет собой одну из важных медико-социальных проблем, с которой столкнулось человечество за последний год [21, 22]. Как известно, большинство пациентов с COVID-19 имеют благоприятный прогноз, однако у части больных инфекция может приводить к тяжелым системным поражениям, требующим госпитализации, и нередко быть сопряженной с высокой летальностью [23]. К настоящему моменту уже известны факторы, увеличивающие риск тяжести течения и смертности при COVID-19, к которым относят пожилой возраст, мужской пол, наличие сахарного диабета и/или ожирения, а также сердечно-сосудистые заболевания [22][24]. Дополнительно к этим факторам исследования последних лет показали, что низкий уровень обеспеченности витамином D может представлять собой один из модифицируемых факторов риска развития коронавирусной инфекции COVID-19, а также ухудшающих течение и прогноз заболевания [25, 26]. Увеличение числа больных ОРВИ, так же как и лиц с дефицитом витамина D от юга к северу [27], а также наличие рецепторов к витамину D и экспрессия фермента 1-α гидроксилазы, участвующей в образовании активного гормона D — кальцитриола, в клетках иммунной системы явились предпосылкой для исследования иммуномодулирующих свойств данного нутриента [11][12]. Самые первые данные об уровне 25(ОН)D крови у больных COVID-19 поступили из Китая, где была описана ассоциация между низким уровнем обеспеченности витамином D и тяжестью/исходами заболевания [28][29]. В последующем появились работы, свидетельствующие о наличии обратной связи между концентрацией 25(OH)D в сыворотке крови и тяжестью COVID-19, а также смертностью больных [30–32].

Анализируя данные самого крупного на сегодня исследования, проведенного в США и объединившего информацию о результатах ПЦР-теста на SARS-CoV-2 и уровней обеспеченности витамином D более чем у 190 000 человек, можно видеть зависимость количества положительных результатов ПЦР-теста от показателя концентрации 25(ОН)D в сыворотке крови [16]. Так, авторами установлено, что при концентрации 25(OH)D менее 20 нг/мл положительный тест на SARS-CoV-2 имели 39 120 пациентов, что составило 12,5% (95% ДИ 12,2–12,8%). Концентрация 25(ОН)D 30–34 нг/мл была ассоциирована с несколько меньшим числом инфицированных: 27 870 пациентов, что соответствовало 8,1% (95% ДИ 7,8–8,4%). Наконец, при уровне 25(ОН)D >55 нг/мл только 12 321 пациент имел положительный тест (5,9%; 95% ДИ 5,5–6,4%). Таким образом, исследователи продемонстрировали, что наибольшее количество инфицированных наблюдалось при концентрации 25(OH)D в крови менее 20 нг/мл, а при значении показателя более 50 нг/мл положительный тест на SARS-CoV-2 встречался на 40% реже, чем у лиц с дефицитом витамина D.

Сопоставление с другими публикациями

В отличие от зарубежных данных результаты проведенного исследования, как и более ранние отечественные работы [4][33], не выявили закономерностей во встречаемости недостатка и дефицита витамина D в зависимости от географического расположения региона и показали наличие одинаково низких уровней 25(ОН)D во всех ФО, в которых проживали обследованные. Однако, как видно из представленных данных, в южных регионах (Южный, Приволжский, Дальневосточный и Северо-Кавказский ФО) количество лиц с дефицитом витамина D было все-таки несколько больше, чем в центральных (Центральный и Уральский ФО) и северных (Северо-Западный и Сибирский ФО) регионах, что, вероятно, связано с большей информированностью и более частым назначением профилактических доз колекальциферола в более удаленных от экватора районах. В то же время было установлено, что дефицит витамина D чаще встречался среди лиц старше 75 лет, а нормальный уровень обеспеченности витамином D был характерен для половины лиц моложе 18 лет, что согласуется с данными ранее проведенных исследований [1–3]. Однако отсутствие информации о приеме препаратов витамина D не позволяет нам уточнить наличие недостаточного или нормального уровня обеспеченности данным нутриентом в зависимости от приема колекальциферола.

Клиническая значимость результатов

Анализ данных об инфицированности COVID-19 среди жителей восьми ФО на основании результатов ПЦР-тестов одной из крупнейших сетевых лабораторий (ООО «НПФ «ХЕЛИКС») показал отсутствие различий в значениях количества позитивных тестов на SARS-Co-2 во всех регионах. В связи с малым количеством положительных тестов у жителей Дальневосточного региона с известным уровнем обеспеченности витамина D на период 31.10.2020 г. мы не можем в полной мере судить о вкладе дефицита витамина D в инфицированность COVID-19 на Дальнем Востоке. Однако, как видно из представленных данных по другим регионам, включая южные, мы не выявили зависимости количества позитивных тестов на SARS-CoV-2 от уровня 25(ОН)D крови. Таким образом, в отличие от данных, опубликованных H.W. Kaufman и соавт., инфицированность COVID-19 в РФ не ассоциирована с уровнем обеспеченности витамином D, хотя дефицит этого важного для здоровья нутриента сохраняется во всех регионов и более часто диагностируется у лиц старше 75 лет. Учитывая факторы риска развития и течения коронавирусной инфекции, представляется чрезвычайно важным уделять большее внимание вопросам профилактики и лечения, особенно у этой возрастной группы.

Ограничения исследования

Отсутствие информации о приеме, дозах и длительности приема препаратов витамина D. Отсутствие данных о наличии заболеваний, включая патологию желудочно-кишечного тракта, почек, которые могли повлиять на значения 25(ОН)D у изучаемой популяции.

Направления дальнейших исследований

В планах проведение интервенционных исследований для оценки вклада терапии стандартными и насыщающими дозами колекальциферола в профилактику инфицирования SARS-CoV-2 медицинских работников, работающих в «красной» зоне, а также в снижение тяжести и летальности больных COVID-19 при добавлении к стандартной терапии.

## ЗАКЛЮЧЕНИЕ

Таким образом, результаты исследования еще раз подтвердили высокую распространенность недостатка и дефицита витамина D в РФ, сохраняющуюся в период пандемии новой коронавирусной инфекции и наиболее часто представленную в группах лиц старше 75 лет. Однако нами не была выявлена зависимость между уровнем обеспеченности витамином D и числом положительных ПЦР-тестов к SARS-CoV-2 ни в одном из регионов проживания.

## ДОПОЛНИТЕЛЬНАЯ ИНФОРМАЦИЯ

Источники финансирования. Государственное задание МЗ РФ. Рег №: 121031100284-7.

Конфликт интересов. Авторы декларируют отсутствие явных и потенциальных конфликтов интересов, связанных с содержанием настоящей статьи.

Участие авторов. Каронова Т.Л. — существенный вклад в концепцию и дизайн исследования, в получение, анализ данных и интерпретацию результатов; написание статьи или внесение в рукопись существенной правки с целью повышения научной ценности статьи; Андреева А.Т. — существенный вклад в концепцию исследования, анализ данных и интерпретацию результатов, написание статьи; Головатюк К.А. — существенный вклад в получение данных, написание статьи; Быкова Е.С. — существенный вклад в получение данных, написание статьи; Скибо И.И. — существенный вклад в концепцию исследования, получение данных, внесение в рукопись важной правки с целью повышения научной ценности статьи; Гринева Е.Н. — существенный вклад в концепцию исследования, внесение в рукопись важной правки с целью повышения научной ценности статьи; Шляхто Е.В. — существенный вклад в концепцию исследования, внесение в рукопись важной правки с целью повышения научной ценности статьи. Все авторы одобрили финальную версию статьи перед публикацией, выразили согласие нести ответственность за все аспекты работы, подразумевающую надлежащее изучение и решение вопросов, связанных с точностью или добросовестностью любой части работы.

Благодарности. ООО «НПФ «ХЕЛИКС» за предоставленные данные. Пантелеевой Ольге Игоревне, специалисту по медицинской статистике; e-mail: oipanteleeva@mail.ru.
